# The Prevalence of Nine Genetic Disorders in a Dog Population from Belgium, the Netherlands and Germany

**DOI:** 10.1371/journal.pone.0074811

**Published:** 2013-09-19

**Authors:** Bart J. G. Broeckx, Frank Coopman, Geert E. C. Verhoeven, Wim Van Haeringen, Leanne van de Goor, Tim Bosmans, Ingrid Gielen, Jimmy H. Saunders, Sandra S. A. Soetaert, Henri Van Bree, Christophe Van Neste, Filip Van Nieuwerburgh, Bernadette Van Ryssen, Elien Verelst, Katleen Van Steendam, Dieter Deforce

**Affiliations:** 1 Laboratory of Pharmaceutical Biotechnology, Faculty of Pharmaceutical Sciences, Ghent University, Ghent, Belgium; 2 Holbigenetics NV, Waarschoot, Belgium; 3 Department of Medical Imaging and Small Animal Orthopaedics, Faculty of Veterinary Medicine, Ghent University, Merelbeke, Belgium; 4 Dr. Van Haeringen Laboratorium b.v., Wageningen, The Netherlands; 5 Department of Medicine and Clinical Biology of Small Animals, Faculty of Veterinary Medicine, Ghent University, Merelbeke, Belgium; 6 Faculty of Applied Bio-engineering, University College Ghent, Melle, Belgium; University of Sydney, Australia

## Abstract

The objective of this study was to screen a dog population from Belgium, the Netherlands and Germany for the presence of mutant alleles associated with hip dysplasia (HD), degenerative myelopathy (DM), exercise-induced collapse (EIC), neuronal ceroid lipofuscinosis 4A (NCL), centronuclear myopathy (HMLR), mucopolysaccharidosis VII (MPS VII), myotonia congenita (MG), gangliosidosis (GM1) and muscular dystrophy (Duchenne type) (GRMD). Blood samples (K3EDTA) were collected for genotyping with Kompetitive Allele Specific PCR (n = 476). Allele and genotype frequencies were calculated in those breeds with at least 12 samples (n = 8). Hardy-Weinberg equilibrium was tested. Genetic variation was identified for 4 out of 9 disorders: mutant alleles were found in 49, 15, 3 and 2 breeds for HD, DM, EIC and NCL respectively. Additionally, mutant alleles were identified in crossbreeds for both HD and EIC. For HD, DM, EIC and NCL mutant alleles were newly discovered in 43, 13, 2 and 1 breed(s), respectively. In 9, 2 and 1 breed(s) for DM, EIC and NCL respectively, the mutant allele was detected, but the respective disorder has not been reported in those breeds. For 5 disorders (HMLR, MPS VII, MG, GM1, GRMD), the mutant allele could not be identified in our population. For the other 4 disorders (HD, DM, EIC, NCL), prevalence of associated mutant alleles seems strongly breed dependent. Surprisingly, mutant alleles were found in many breeds where the disorder has not been reported to date.

## Introduction

The reduction of genetic disorders remains an important goal for both veterinarians and breeders [1]. It is the responsibility of the scientific community to provide detailed information regarding the existence, application and importance of diagnostic genetic tests that have been developed [1]–[3]. An important step in this process is to evaluate the prevalence of disorders and mutant alleles in the population. This information is needed to provide proper breeding advice. So far, only a few studies have been conducted to identify allele frequencies in a canine population [3]–[6]. The presence of mutant alleles in different breeds with each having their specific genetic background, might provide interesting (clinical) information for both the animal and human population as many canine disorders are animal models for human genetic disorders.

This study reports on the prevalence of mutant alleles associated with 9 canine genetic disorders (Table 1) that influence the neuronal and/or musculoskeletal system: hip dysplasia (HD), degenerative myelopathy (DM), exercise-induced collapse (EIC), neuronal ceroid lipofuscinosis 4A (NCL), centronuclear myopathy (HMLR), mucopolysaccharidosis VII (MPS VII), myotonia congenita (MG), gangliosidosis (GM1) and muscular dystrophy (Duchenne type) (GRMD)). The tests were performed in a dog population from Belgium, the Netherlands and Germany. Eight out of these 9 disorders are animal models for similar conditions in humans. The mutations predicted to cause HD, DM, EIC and NCL, were detected in a wide variety of breeds.

**Table 1:** Overview of 9 disorders tested with their corresponding chromosome number, the mutation, the effect, the inheritance, the breeds where the mutation has been reported before and the animal model.

Disorder	Gene	CFA	Mutation		Effect	inheritance	Breeds with mutation reported so far	Similar human disease	Reference
Hip dysplasia (HD)	*Fibrillin 2 (FBN2)* (Gene ID: 481491)	11	intronic (3 SNPs+deletion)	GAT>AGC	?	MP	Labrador Retriever, Border Collie, German Shepherd Dog, Golden Retriever, Newfoundland, Rottweiler, Great Dane	Congenital Contractural Arachnodactyly	[9]
Degenerative myelopathy (DM)	*Superoxide dismutase 1 (SOD1)* (Gene ID: 403559)	31	exon (1 SNP)	G>A	MS	AR^2^	German Shepherd Dog, Boxer, Rhodesian Ridgeback, Chesapeake Bay Retriever, Pembroke Welsh Corgi	Amyotrophic lateral sclerosis	[8]
Exercise-induced collapse (EIC)	*Dynamin 1 (DNM1)* (Gene ID: 491319)	9	exon (1 SNP)	G>T	MS	AR^2^	Labrador Retriever, Chesapeake Bay Retriever, Curly-coated Retriever, Boykin Spaniel, Pembroke Welsh Corgi	–	[11]
Neuronal ceroid lipofuscinosis 4A (NCL)	*Arylsulfatase G (ARSG)* (Gene ID: 480460)	9	exon (1 SNP)	G>A	MS	AR^2^	American Staffordshire Terrier	Kufs disease	[7]
Centronuclear myopathy (HMLR)	*Protein tyrosine phosphatase-like (PTPLA)* (Gene ID: 574011)	2	exon (SINE)	SINE	MS	AR	Labrador Retriever	Human centronuclear myopathy	[12]
Mucopolysaccharidosis VII (MPS VII)	*Beta-glucuronidase (GUSB)* (Gene ID: 403831)	6	exon (1 SNP)	G>A	MS	AR	German Shepherd Dog	Mucopolysaccharidosis VII	[13]
	*Beta-glucuronidase (GUSB)* (Gene ID: 403831)	6	exon (1 SNP)	C>T	MS	AR	Brazilian Terrier	Mucopolysaccharidosis VII	[10]
Myotonia congenita (MG)	*Chloride channel, voltage sensitive 1 (CLCN1)* (Gene ID: 403723)	16	exon (1 SNP)	C>T	MS	AR	Miniature Schnauzer	Generalized myotonia (Beckers disease)	[14]
Gangliosidosis (GM1)	*Beta-galactosidase (GLB1)* (Gene ID: 403873)	23	exon (deletion)	C	FS^1^	AR	Shiba	Gangliosidosis	[16]
Muscular dystrophy (Duchenne) (GRMD)	*Dystrophin (DMD)* (Gene ID: 606758)	X	intron (1 SNP)	A>G	ES	XR	Golden Retriever	Duchenne Muscular Dystrophy	[15]

CFA = chromosome number; AR = autosomal recessive, XR = X-linked recessive, MP = multifactorial; MS = missense, SP = splice variant, FS = frame shift, ES = exon skipping, SINE = short interspersed element, SNP = single nucleotide polymorphism;^ 1^ leading to premature termination, ^2^ reduced penetrance.

## Materials and Methods

### Ethics Statement

Approval from the local ethical (Faculty of Veterinary Medicine, Ghent University, Belgium) and deontological (Federal Public Service Health, Food Chain Safety and Environment, Brussels, Belgium) committees was granted (EC2010_171 and EC2011_193). All efforts were made to minimize suffering. Informed consent was obtained from owners of dogs before enrollment in the study.

### Sample Collection

Blood samples (K3EDTA) were collected for a genetic database to study HD. Veterinarians from Belgium, the Netherlands and Germany were asked to take a blood sample from every dog that had a hip radiograph taken. Reasons for performing the procedure varied from screening purposes (breeding and assistance dogs) to dogs with clinical complaints (with HD in the differential diagnosis). No prerequisites were made regarding breed, sex and age.

Irrespective of breed, samples (n = 476) were tested for the presence of mutations associated with 9 disorders (HD, DM, EIC, NCL, HMLR, MPS VII, MG, GM1 and GRMD). Breeds where the mutant allele has already been reported, can be found in Table 1. A summary of breeds and samples per breed can be found in Table 2. Additionally, a mixed breed group of 28 dogs was tested.

**Table 2:** Breed and samples per breed tested.

Breed	n	%	Breed	n	%
Airedale Terrier	2	0.4	Gordon Setter	1	0.2
Akita	1	0.2	Great Dane	1	0.2
American Bulldog	1	0.2	Hovawart	1	0.2
American Cocker Spaniel	1	0.2	Hungarian Vizsla	7	1.5
American Staffordshire Terrier	18	3.8	Jack Russell Terrier	2	0.4
Anatolian Shepherd Dog	2	0.4	Labrador Retriever	75	15.8
Appenzeller Sennenhund	1	0.2	Laekenois	1	0.2
Australian Kelpie	1	0.2	Large Munsterlander	1	0.2
Australian Shepherd	6	1.3	Leonberger	4	0.8
Basset Hound	1	0.2	Malinois	7	1.5
Berger de Picardie	2	0.4	Maltese	1	0.2
Bernese Mountain Dog	20	4.2	Mastino Napoletano	1	0.2
Blue Picardy Spaniel	1	0.2	Miniature Pinscher	1	0.2
Boerboel	1	0.2	Munsterlander	1	0.2
Border Collie	29	6.1	Nederlandse Schapendoes	1	0.2
Bouvier des Flandres	3	0.6	Newfoundland	4	0.8
Boxer	15	3.2	Nova Scotia Duck Tolling Retriever	3	0.6
Briard	1	0.2	Rhodesian Ridgeback	1	0.2
Bull Terrier	1	0.2	Rottweiler	7	1.5
Cavalier King Charles Spaniel	4	0.8	Saarlooswolfhond	2	0.4
Collie Rough	2	0.4	Saint Bernard Dog	3	0.6
Dalmatian	2	0.4	Samoyed	1	0.2
Dobermann	2	0.4	Shar Pei	4	0.8
Dogo Argentino	1	0.2	Shetland Sheepdog	1	0.2
Dogue de Bordeaux	5	1.1	Shiba	3	0.6
Dwergschnauzer	1	0.2	Siberian Husky	2	0.4
English Bulldog	4	0.8	Spanish Water Dog	12	2.5
English Cocker Spaniel	2	0.4	Springer Spaniel^a^	1	0.2
English Setter	2	0.4	Stabyhoun	3	0.6
English Springer Spaniel	1	0.2	Standard Poodle	2	0.4
Epagneul Breton	2	0.4	Tervueren	2	0.4
Flat Coated Retriever	8	1.7	Tibetan Mastiff	1	0.2
French Bulldog	3	0.6	Weimaraner	4	0.8
German Shepherd Dog	73	15.3	White Swiss Shepherd Dog	5	1.1
Golden Retriever	62	13.0	Wire-Haired Pointing Griffon Korthals	1	0.2
			Total	476	

a = not specified whether English or Welsh Springer Spaniel.

### Genotyping

Genomic DNA was isolated using routine procedures. For blood samples, 10 µl of blood was washed 3 times with 150 µl of a Tris-HCL based buffer. The procedure was performed with the use of robotic equipment. The cell pellet was lysed with Proteinase K (0.5 units for 45 minutes at 56°C followed by heat inactivation at 95°C for 5 minutes).

Genotyping was conducted using KASP (http://www.kbioscience.co.uk), a competitive allele specific polymerase chain reaction system, according to the manufacturer’s instructions. Primer sequences were based on literature [7]–[16] and transformed to be compatible with KASP. All tests are routinely run at the dr. Van Haeringen Laboratorium (Wageningen, the Netherlands).

### Statistical Analysis

Breed specific prevalence was analyzed in 8 breeds for which at least 12 samples were available (German Shepherd Dog, Labrador Retriever, Golden Retriever, Border Collie, Bernese Mountain Dog, American Staffordshire Terrier, Boxer, Spanish Water Dog). Hardy – Weinberg equilibrium (HWE) was tested with an online calculator (www.tufts.edu/~mcourt01/Documents). Data are available on request.

## Results

No variation was found in any of the breeds for the mutations putatively responsible for 5 of the 9 disorders (HMLR, MPS VII, MG, GM1, GRMD). The mutations predicted to cause HD, DM, EIC and NCL, were observed in a wide variety of breeds (49, 15, 3 and 2 breeds, respectively). Breeds where mutant alleles were found are listed in Tables 3, 4, 5 and 6 for HD, DM, EIC and NCL, respectively. For HD, DM, EIC and NCL mutant alleles were newly discovered in 43, 13, 2 and 1 breed(s), respectively. The mutant alleles for HD and EIC were also identified in mixed breed dogs. For 25 of the 28 crossbreds, the parental breeds were known and we have shown these breeds to possess the respective mutant allele.

**Table 3:** Breeds where mutant alleles for hip dysplasia were found and breed specific prevalence for breeds with at least 12 samples.

	NN	NA	AA	Total	HWE	q (%)
	n (%)	n (%)	n (%)	n (%)	P-value	
American Staffordshire Terrier	4 (24)	9 (53)	4 (24)	17 (100)	–	50
Bernese Mountain Dog	7 (35)	12 (60)	1 (5)	20 (100)	–	35
Border Collie	25 (89)	3 (11)	0 (0)	28 (100)	–	5
Boxer	15 (100)	0 (0)	0 (0)	15 (100)	–	0
German Shepherd Dog	34 (47)	34 (47)	4 (6)	72 (100)	0.225	29
Golden Retriever	10 (16)	31 (51)	20 (33)	61 (100)	0.728	58
Labrador Retriever	32 (45)	21 (30)	18 (25)	71 (100)	0.001	40
Spanish Water Dog	4 (33)	8 (67)	0 (0)	12 (100)	–	33
Others:	Airedale Terrier, Appenzeller Sennenhund, Australian Shepherd, Blue Picardy Spaniel, Boerboel, Bouvier des Flanders, Briard, Bull Terrier, Cavalier King Charles Spaniel, Collie Rough, Dalmatian, Dobermann, Dogo Argentino, Dogue de Bordeaux, English Bulldog, English Cocker Spaniel, English Setter, English Springer Spaniel, Epagneul Breton, Flat Coated Retriever, Gordon Setter, Hovawart, Hungarian Vizsla, Laekenois, Large Munsterlander, Leonberger, Malinois, Maltese, Mastino Napoletano, Miniature Pinscher, Newfoundland, Nova Scotia Duck Tolling Retriever, Rhodesian Ridgeback, Rottweiler, Saarlooswolfhond, Saint Bernard Dog, Shetland Sheepdog, Siberian Husky, Springer Spaniel^a^, Stabyhoun, Standard Poodle, Weimaraner, White Swiss Shepherd Dog					

NN = 2 normal alleles, NA = heterozygous, AA = 2 mutant alleles, HWE = Hardy-Weinberg equilibrium, q = mutant allele frequency, % = percent of dogs belonging to specific category, – = not applicable, ^a^ = not specified whether English or Welsh Springer Spaniel.

**Table 4:** Breeds where mutant alleles for degenerative myelopathy were found and breed specific prevalence for breeds with at least 12 samples.

	NN	NA	AA	Total	HWE	q (%)
	n (%)	n (%)	n (%)	n (%)	P-value	
American Staffordshire Terrier	18 (100)	0 (0)	0 (0)	18 (100)	–	0
Bernese Mountain Dog	12 (60)	7 (35)	1 (5)	20 (100)	–	23
Border Collie	27 (96)	1 (4)	0 (0)	28 (100)	–	2
Boxer	13 (87)	2 (13)	0 (0)	15 (100)	–	7
German Shepherd Dog	53 (73)	18 (25)	2 (3)	73 (100)	–	15
Golden Retriever	62 (100)	0 (0)	0 (0)	62 (100)	–	0
Labrador Retriever	74 (100)	0 (0)	0 (0)	74 (100)	–	0
Spanish Water Dog	12 (100)	0 (0)	0 (0)	12 (100)	–	0
Others:	Airedale Terrier, Australian Shepherd, Cavalier King Charles Spaniel, Collie Rough, Dobermann, Dogo Argentino, Saarlooswolfhond, Shetland Sheepdog, Stabyhoun, Standard Poodle, White Swiss Shepherd Dog					

NN = 2 normal alleles, NA = heterozygous, AA = 2 mutant alleles, HWE = Hardy-Weinberg equilibrium, q = mutant allele frequency, % = percent of dogs belonging to specific category, – = not applicable.

**Table 5:** Breeds where mutant alleles for exercise-induced collapse were found and breed specific prevalence for breeds with at least 12 samples.

	NN	NA	AA	Total	HWE	q (%)
	n (%)	n (%)	n (%)	n (%)	P-value	
American Staffordshire Terrier	18 (100)	0 (0)	0 (0)	18 (100)	–	0
Bernese Mountain Dog	18 (100)	0 (0)	0 (0)	18 (100)	–	0
Border Collie	25 (100)	0 (0)	0 (0)	25 (100)	–	0
Boxer	12 (100)	0 (0)	0 (0)	12 (100)	–	0
German Shepherd Dog	69 (100)	0 (0)	0 (0)	69 (100)	–	0
Golden Retriever	51 (100)	0 (0)	0 (0)	51 (100)	–	0
Labrador Retriever	30 (46)	16 (25)	19 (29)	65 (100)	<0.001	42
Spanish Water Dog	12 (100)	0 (0)	0 (0)	12 (100)	–	0
Others:	Hungarian Vizsla, English Cocker Spaniel					

NN = 2 normal alleles, NA = heterozygous, AA = 2 mutant alleles, HWE = Hardy-Weinberg equilibrium, q = mutant allele frequency, % = percent of dogs belonging to specific category, – = not applicable.

**Table 6:** Breeds where mutant alleles for neuronal ceroid lipofuscinosis 4A were found and breed specific prevalence for breeds with at least 12 samples.

	NN	NA	AA	Total	HWE	q (%)
	n (%)	n (%)	n (%)	n (%)	P-value	
American Staffordshire Terrier	14 (82)	3 (18)	0 (0)	17 (100)	–	9
Bernese Mountain Dog	19 (100)	0 (0)	0 (0)	19 (100)	–	0
Border Collie	26 (100)	0 (0)	0 (0)	26 (100)	–	0
Boxer	13 (100)	0 (0)	0 (0)	13 (100)	–	0
German Shepherd Dog	71 (100)	0 (0)	0 (0)	71 (100)	–	0
Golden Retriever	57 (100)	0 (0)	0 (0)	57 (100)	–	0
Labrador Retriever	70 (100)	0 (0)	0 (0)	70 (100)	–	0
Spanish Water Dog	12 (100)	0 (0)	0 (0)	12 (100)	–	0
Others:	Bull Terrier					

NN = 2 normal alleles, NA = heterozygous, AA = 2 mutant alleles, HWE = Hardy-Weinberg equilibrium, q = mutant allele frequency, % = percent of dogs belonging to specific category, - = not applicable.

For 8 breeds, breed specific prevalence of alleles were reported (Table 3–6). Comparisons with previous reports could only be made for EIC in the Labrador Retriever: our population contained a very high number of genetically affected dogs (Table 7) [6]. HWE could be tested for HD in the German Shepherd Dog and the Golden Retriever (no significant deviation). For the Labrador Retriever, genotype frequencies for HD and EIC both deviated significantly from HWE (p≤0.001). The other breeds and disorders were not tested due to low number of samples and/or absence of variation.

**Table 7:** Comparisons of genotype frequencies for exercise-induced collapse in the Labrador Retriever.

			Reference populations [6]	
Predicted Phenotype	Our population	Est. Freq (HWE)	Source: Public	Source: researchers
	% (n)	% (n)	% (n)	% (n)
Healthy (homo)	46.2 (30)	34.2 (22.2)	52.9 (4826)	59.2 (509)
Healthy (hetero)	24.6 (16)	48.6 (31.6)	37.2 (3392)	34.5 (297)
Affected	29.2 (19)	17.2 (11.2)	9.9 (907)	6.3 (54)
Total	100 (65)	100 (65)	100 (9125)	100 (860)

The reference population consists of 2 different subsets based on collection method. Source: Public = based on request by the owner to perform genetic testing for EIC, Soure: researchers = researchers went to several competitions and took samples from every dog. Est. Freq (HWE) = estimated frequencies under Hardy-Weinberg equilibrium.

## Discussion

Since its first report in 1935, intensive research has focused on hip dysplasia (HD), one of the most frequent orthopedic disorder in dogs [17]. HD can be found in a wide variety of breeds [18], [19]. Recently, a haplotype in the *Fibrillin 2 (FBN2)* gene has been reported to be associated with this highly prevalent, multifactorial disorder [9]. Fibrillins are components of extracellular microfibrils and have both a structural and a regulatory function [20]. The mutant *AGC haplotype* was identified in 49 different breeds in the population under study (Table 3). In 44 breeds, this mutant allele was reported for the first time. We identified the *AGC haplotype* as the only allele in 10 breeds (Cavalier King Charles Spaniel (n = 4), English Setter (n = 2), English Springer Spaniel (n = 1), Gordon Setter (n = 1), Laekenois (n = 1), Mastino Napoletano (n = 1), Rhodesian Ridgeback (n = 1), Saarlooswolfhond (n = 2), Siberian Husky (n = 2), Standard Poodle (n = 2)), however few conclusions can be made as the sample count is low (n = 17 overall). One additional dog was homozygous for the mutant allele, but as the breed was not correctly specified (Springer Spaniel without mentioning English or Welsh Springer Spaniel), this sample was excluded.

Degenerative myelopathy (DM) is characterized by progressive ataxia and upper motor neuron spastic paresis. The majority of dogs with DM start to develop symptoms from 5 years of age [21]. Diagnosis is not straightforward when patients are alive. Based on clinical symptoms and exclusion of other disorders (for example intervertebral disc disease, spinal cord neoplasia), DM can be the most likely etiology, but formal diagnosis can only be achieved post-mortem on histopathology [21]. Only for a subset of those breeds in which DM has been reported clinically, this disorder has been confirmed on histopathology. In 2009, a causal mutation was discovered in the *superoxide dismutase 1 (SOD1)* gene for 5 breeds (Table 1) with an age-dependent incomplete penetrance [8]. This gene encodes a free radical scavenger [21]. We identified the causal mutation in 15 breeds. In 2 breeds (Standard Poodle and Bernese Mountain Dog) the disorder was confirmed both clinically and on histopathology while in another 2 breeds (Border Collie and Collie Rough) the diagnosis of DM was made only on clinical examination [21]. We report the presence of the mutant allele in these 4 breeds and in an additional 9 breeds, in which the disorder has not yet been reported. We also confirmed the presence of the mutant allele in the German Shepherd Dog and Boxer (Table 4). DM has been clinically reported in the Labrador Retriever and confirmed by histopathology in the Golden Retriever, but the mutation has not yet been reported in these breeds [21]. In our population of respectively 74 and 62 individuals, we also did not identify the *SOD1* mutation. The mutant allele might be infrequently present in the population or a different mutation might be responsible for the same disorder, as recently reported [22].

Dogs with exercise-induced collapse (EIC) develop incoordination of the hind limbs, paraparesis and/or tetraparesis and have an increased body temperature after strenuous exercise. A mutation in the *dynamin 1 (DNM1)* gene was found to be responsible for this disorder and an incomplete penetrance (influenced by the level of physical activity) was suggested [11]. *DNM1* is important in neuronal synaptic vesicle recycling, especially during high levels of activity [23]. This disorder is reported mainly in Labrador Retrievers, but presence of the mutant allele has also been reported in other retriever breeds [6], [11]. We report on the presence of the mutant allele for the first time in one English Cocker Spaniel and one Hungarian Vizsla (Table 5). The prevalence of EIC in our Labrador population was higher than in a previous report (Table 7) [6]. The sample source seems to affect the percentage of affected dogs: when samples were collected from Labrador Retrievers in dog shows, field trials and from local pet owners in Canada and the United States, the prevalence of affected dogs was relatively low and in agreement with HWE expectations [6]. The opposite was true for dogs that were specifically tested for EIC and those results were in agreement with the results from our study, although we did not specifically select for those dogs. In our population of Labrador Retrievers, genotype frequencies for both HD and EIC deviate significantly from HWE. This might be a reflection of non-random sampling or selection [4]. For HD, non-random sampling can be expected since our dogs were initially collected to study this disorder. For EIC, the reason for rejection of the HWE is not clear. For both HD and EIC, our results tend to overestimate the number of affected dogs based on HWE. A similar result was found in the pet population in a previous study (26.9 versus 29.2%) [6].

Neuronal ceroid lipofuscinosis 4A (NCL) has been reported in American Staffordshire Terriers with progressive ataxia [24]. The causal mutation was discovered in 2010 in the *Arylsulfatase G (ARSG)* gene and an incomplete penetrance was suggested [7]. *ARSG* encodes for a lysosomal enzyme [25]. The mutant allele has a prevalence of approximately 9% in our 17 American Staffordshire Terriers which is less than the frequency expected based on a previous study [24]. We report the presence of the same mutation in the Bull Terrier, a breed where NCL has not been reported (Table 6). No dogs were homozygous for the mutant allele in the population studied.

For the other 5 disorders (HMLR, MPS VII, MG, GM1, GRMD) the mutant alleles were not detected in our population. Non-detection of the mutant allele might indicate that the allele is absent, that it is present but at a low frequency and that the number of samples tested was too low. For HMLR (Labrador Retriever, n = 66) and MPS VII (German Shepherd Dog, n = 67), we had, with the numbers of animals tested, a 99% chance of detecting every allele with a minor allele frequency (MAF) of at least 3.5% [26]. For GRMD (Golden Retriever, n = 19), we had a 99% chance of detecting alleles with a frequency of at least 11.5% [26]. For MG, GM1 and MPS VII (in the Brazilian Terrier), the sample size was too small to make any conclusions regarding the absence of the mutant allele [26]. The allele frequency for HMLR has previously been investigated [4]. In that study, the mutant allele frequency was very low (1.8% or 0.47%). To be able to detect all alleles with a MAF of 1% with a 99.9% probability, 344 samples would be needed. Since our sample count was much lower, it cannot be concluded that any of these 5 mutant alleles are completely absent.

For DM, EIC and NCL breeding advice can be given based on our population study. For some disorders, the mutant allele frequency is quite high in certain breeds (>40% in Labrador Retriever for EIC). However, because of their recessive nature, a relatively fast reduction of both the mutant allele frequency and affected dogs can be achieved based on genetic tests. We propose to exclude certain genotypic combinations of dogs from mating, rather than excluding individuals, especially if high mutant allele frequencies are found. For autosomal recessive diseases, dogs homozygous for the mutant allele should not be combined with each other or with heterozygous dogs. However, they can still be used for breeding, but require a mating combination involving only homozygous wild type animals to reduce the number of affected dogs. As heterozygous dogs can be used the same way, no dogs need to be excluded. As essentially every dog can still be used for breeding, the clinical outcome of disorder can be prevented without excessive exclusion of carrier or genetically affected dogs from the breeding population. Reduction of the prevalence of HD based only on *FBN2* will be more difficult since it is a multifactorial polygenetic disorder.

Surprisingly, mutant alleles for 3 autosomal recessive disorders (DM, EIC and NCL) were found in 9, 2 and 1 breed(s) respectively where the disorder has not been clinically reported. The most plausible explanation might be that the disorder just has not been recognized in those breeds. A second explanation is the influence of the breed specific genetic background: the effect of mutations might be different in different breeds. This has been reported in Drosophila and the mouse [27], [28]. In a meta-analysis in humans where studies on a wide variety of diseases and genes were compared, opposite effects between races were found, but none of them significant [29]. To the authors’ knowledge, this phenomenon has not yet been reported in dogs.

This study reports on the presence of mutant alleles for 9 disorders in a wide variety of dog breeds. Veterinarians and dog breeders should be aware that mutations are present in breeds even where the disorder has not been reported. Dogs from non-suspected breeds that show comparable symptoms to the disorders reported in this and other studies should be genotyped and results should be reported in order to create a reliable database. Ideally, phenotypical information for every disorder should be included in this database. As this is not available for all diseases in our database, this is a major limitation to this study.
